# Antipsychotic-Induced Weight Gain and Clinical Improvement: A Psychiatric Paradox

**DOI:** 10.3389/fpsyt.2020.560006

**Published:** 2020-11-03

**Authors:** Clemente Garcia-Rizo

**Affiliations:** Barcelona Clinic Schizophrenia Unit, Hospital Clinic de Barcelona, Institute of Neuroscience, University of Barcelona, Centre for Biomedical Research in Mental Health, August Pi i Sunyer Biomedical Research Institute, Barcelona, Spain

**Keywords:** antipsychotic induced weight gain, clinical improvement, schizophrenia, psychosis, clozapine, olanzapine

Almost a hundred and thirty years ago, Emil Kraepelin described important weight changes in patients diagnosed with dementia praecox during the acute state of psychosis outbreaks (1). Kraepelin suggested that if the patient remained under severe psychotic symptomatology (incoherent, uninterested) despite weight gain, there was a great probability that the disease had reached a final and unfavorable prognosis. However, it was not the first reference to the topic, as Nasse (2), reviewing the existing literature at the time, stated that since the use of Esquirol, an axiom had been described in the realm of mental health, “the healing on the insane began with improved nutrition” (2).

Twenty years later Eugenie Bleuler in his seminal book “Dementia praecox oder Gruppe der Schizophrenien” described that body weight of patients underwent irregular and severe variations from which no cause had been identified (being sometimes as much as 25 Kg), suggesting that it could not be considered as a compensation mechanism after the stressful circumstances of a psychotic outbreak (3). He also reported again that weight increase without clinical improvement was a sign of bad prognosis.

Nevertheless the link faded with the introduction of chlorpromazine in 1952 like other medical conditions [i.e., glucose disturbances (4)]. However, several articles on patients treated with chlorpromazine during the subsequent decades maintained the interest in weight gain and clinical outcome (5–8). Planansky, after acknowledging the possible concomitant effect of weight gain and clinical amelioration, suggested that it was due to more food consumption after its clinical improvement (7). Other studies included different pharmaceutical agents such as perphenazine and phenelzine. Singh, in a longitudinal study with perphenazine, showed a correlation between weight gain and clinical improvement, suggesting weight gain as a useful predictor of an antipsychotic response (9). Holden, in another longitudinal study with patients diagnosed with schizophrenia evaluated in periods of 8 weeks of active treatment (thioridazine, chlordiazepoxide, and a combination of both and placebo) over 14 months, described clinical improvement along with weight gain (10). Nevertheless, further studies evaluating patients treated with clozapine and olanzapine gathered more robust conclusions probably due to its specific metabolic profile (11).

With the reintroduction of clozapine in the market, new studies regarding this association in patients diagnosed with schizophrenia or schizoaffective disorder appeared in the literature. Lamberti, without aiming at it, showed an inverse correlation between clinical symptom severity change and weight gain in 36 inpatients evaluated for 6 months with the brief psychiatric rating scale (BPRS) (12). Leadbetter, in 21 patients evaluated with BPRS for 16 weeks, showed a significant correlation between the reduction in positive and negative symptomatology and weight gain. Indeed, patients who gained more than 10% of their baseline weight showed a significantly greater decrease of the BRPS (13). Bai found gender differences in a sample of 96 patients, where only in females (48%) weight gain was correlated with clinical response (14). Jalenques showed a correlation between long-term efficacy and weigh gain in a longitudinal study involving 15 patients (15). Czobor demonstrated in 38 patients evaluated during 14 weeks that a greater therapeutic response in the general psychopathology score of the positive and negative syndrome scale (PANSS) was associated with greater weight gain (16). Meltzer in a 6-month study (with evaluation at 6 weeks) with 74 patients showed that the percentage change in weight significantly predicted the improvement in the BPRS total and positive symptoms subscale and in the scale for the assessment of negative symptoms (SANS) global score (17). Again Bai, in a retrospective study with 55 patients, showed that 31% who had a significant initial clinical response gained more weight over an 8 year period (18). However, Bustillo did not find any significant association in 19 outpatients treated with clozapine and 20 treated with risperidone over a year of follow-up and evaluations with BPRS and SANS (19). Umbricht in a longitudinal study over 7 years with 82 patients did not find any correlation between weight gain and clinical response (20). And Hummer in 31 patients evaluated with the clinical global impression (CGI) scale with different follow-up periods, did not show any association between weight gain and clinical improvement (21).

The same approach was taken with olanzapine in patients diagnosed with schizophrenia spectrum disorders. Czobor showed a significant correlation between weight gain and improvement in 38 patients (in the general psychopathology, positive, and negative symptoms sub-scores of the PANSS) (16). Garyfallos, in 25 inpatients evaluated during 8 weeks with the PANSS, showed a significant association between clinical improvement and weight gain (22). Ascher-Svanum in two separate studies yielded similar conclusions (23, 24). In a 6 week follow-up study comparing olanzapine, haloperidol, and placebo evaluated with BPRS, 187 patients treated with olanzapine displayed a correlation between weight gain and clinical improvement (23). In another study conducted over a 6 week period, with 1,337 patients treated with olanzapine and evaluated with the BPRS, a significant clinical improvement was observed along with weight gain (24). Ujike in a sample of 164 inpatients evaluated during 8 to 24 weeks displayed similar results (25). Hermes using data from the CATIE trial, evaluated over 72 weeks with the PANSS, described that patients under treatment with olanzapine displayed a significant association between clinical improvement and BMI, however the effect size was too small to be considered clinically substantial (26). Kemp in 107 adolescents evaluated with BPRS over 6 weeks, found a correlation between clinical improvement and weight gain (27). Also Basson, in a longitudinal trial comparing olanzapine with haloperidol over 6 weeks described that weight gain significantly promoted better a clinical outcome (28). However, Agid in a sample of 94 patients treated with olanzapine or ziprasidone over 6 months, described that early weight gain was correlated with less improvement in global functioning (29).

The placebo effect found in some studies is remarkable regarding previous considerations. Ascher-Svanum described that patients under placebo treatment presented a significant association between greater weight gain and greater therapeutic improvement suggesting that weight gain may serve as an important indicator of improved clinical status among acutely ill patients with schizophrenia who do not receive antipsychotic medication (23, 24). Also in Kemp's study the placebo arm showed a trend toward significance between weight gain and clinical improvement (27). Indeed in the study by Holden, findings from the placebo arm showed a correlation between weight loss and clinical deterioration (10).

Despite the paradox that a secondary side effect of antipsychotics such as weight increase might underlie clinical improvement, these mechanisms are worth being discussed. Initially authors underlined this issue suggesting hospital diet, physical inactivity, and the psychological and physical shelter provided by hospitalization as the cause (10). Later several other lines of evidence were proposed: weight gain directly promotes a therapeutic effect, clinical improvement promotes weight gain, and the antipsychotic effect causes both weight gain and a therapeutic effect by an interdependent or dependent pathway (30).

Nevertheless, recent research suggests that the gut-brain axis (GBA), specifically its neurohormones, might behave as a potential pathway underlying both conditions and a key player in promoting weight gain and clinical improvement. The biological effect on the central nervous system of leptin, adiponectin, ghrelin, cholescytokinin (CCK), neuropeptide Y (NPY), glucagon like protein I (GLP-I), and insulin has accumulated further evidence (31). Initially involved in energy balance, appetite, and food intake, its effects have been later extended to synaptic plasticity, cognition, and symptomatology (32).

GBA has been described as a pathophysiological mechanism implicated in antipsychotic-induced weight gain (33, 34), however its implication in clinical symptomatology has received less attention. Venkatasubramanian described variations in serum leptin levels (which are correlated with weight gain) with clinical improvement, specifically negative symptomatology (35) while Konarzewska described a negative correlation between insulin levels and clinical symptomatology (36). Leptin, insulin, and C-peptide levels were identified as reliable biomarkers of relapse in a longitudinal cohort study (37). Indeed research from animal models suggests that modifying the leptin pathway might be a useful target in the treatment of schizophrenia (38). However, in an animal model ghrelin demonstrated worse neuroprotective effects than quetiapine in evaluating stress, anxiety, and spatial memory (39). Also the GLP-1 receptor agonist exenatide, despite being studied for improving metabolic disorders in patients with schizophrenia (40), did not promote any improvement in cognition or psychosocial function in patients (41). Targeting CCK receptors seemed a promising goal in treating the different psychopathological domains of schizophrenia (42), indeed for some time its antipsychotic properties were widely studied (43). Current literature supports the notion that interneurons expressing CCK and NPY receptors modify the dopamine system network promoting changes in anxiety, social interaction, and motor activity (44). Indeed in patients diagnosed with schizophrenia, NPY levels in cerebrospinal fluid were correlated to social function and seemed to predict future outcomes (45). Similar functions have been described for orexin-A, a neuropeptide affecting thermogenesis and energy expenditure which has been described to modify metabolic risk in schizophrenia (46) while high levels have been correlated with fewer negative and disorganized symptoms (47). Although clinical GBA studies focusing on schizophrenia psychopathology remain scarce, they point toward a common pathway involving symptomatology, cognitive function, and clinical outcome.

Considering specifically clozapine or olanzapine, the two pharmacological agents more related with weight gain, they seem to display a direct effect on the hormonal pathways of energy homeostasis (adiponectin and ghrelin) rather than on weight gain (48). Indeed its effect in weight gain seems partially related with specific polymorphisms in the CCK gene (49). Also olanzapine's action on weight gain seems partially attributable to its effect on ghrelin (50) through the activation of the limbic system in response to appetitive stimuli through changes not only in ghrelin but also in leptin and insulin (51).

Overall, previous literature suggests that GBA might be a necessary element in both conditions and so underlie the undeciphered association between weight gain and clinical improvement. However, this association lacks specific studies evaluating the issue and so further studies are required to prove its implication (11).

## Author Contributions

The author confirms being the sole contributor of this work and has approved it for publication.

## Conflict of Interest

CC-R has received honoraria/speaker fees/research and travel support/from Adamed, Alter, Angelini, Janssen-Cilag and Lundbeck.
